# Misinterpretation of Psychiatric Illness in Deaf Patients: Two Case Reports

**DOI:** 10.1155/2018/3285153

**Published:** 2018-06-07

**Authors:** Ethan Anglemyer, Craig Crespi

**Affiliations:** Palm Beach Consortium of Graduate Medical Education/University Hospital and Medical Center, 7201 North University Drive, Tamarac, FL 33321, USA

## Abstract

The Deaf/hard of hearing population is growing rapidly and the medical community is facing a higher demand for this special needs group. The Deaf culture is unique in that spoken word is via sign language. What one person may see as mania or psychosis is actually a norm with Deaf individuals. The fear of the unknown language often creates immediate conclusions that are false. As such, being culturally sensitive becomes a large component of properly assessing a Deaf patient in any psychiatric situation. In the first case, the patient is a 26-year-old prelingually Deaf male, who was placed under an involuntary hold by the emergency room physician for acting erratic and appearing to respond to internal stimuli. The patient was later interviewed with an interpreter and stated he became upset because the staff was not providing him proper care as they lacked an ability to communicate with him. The patient's family was called who corroborated the story and requested he be discharged. Case two presents with a 30-year-old Hispanic male who is also prelingually Deaf. He was admitted involuntary for bizarre behavior and delusions, with a past diagnosis of schizophrenia. Upon interview, the patient endorsed delusions via written language; however, through an ASL-language interpreter he was able to convey a linear and coherent thought process. Caring for special needs patients must be in the repertoire of any trained healthcare professional. Deaf Individuals experience mental illness just like the general population. Symptoms such as auditory hallucinations are not brought up in the same manner and are thought to be a visual construct interpreted by the patient as a vocal expression. It is imperative that these subtle differences are known in order to differentiate out an actual mental illness. In any case where language is a barrier, an interpreter must be present for a thorough assessment. These cases lend further thought into policy reform for Deaf individuals within healthcare.

## 1. Introduction

Approximately 15% of citizens in the United States are either Deaf or hard of hearing. Furthermore 3 out of every 1000 are prelingually Deaf [1]. Prelingually Deaf refers to from time of birth or before verbal language is achieved. Deaf people must use sign language to communicate and express their feelings. Sign language alike with spoken language is not universal and several types exist. The type depends highly on region and country.

Deaf individuals experience mental illness at the same rate as the general population would [2]. This lends the question of “How do we communicate effectively in a medical setting?” In order facilitate this, the clinician must be well versed with the Deaf culture and the diagnostic criteria for psychiatric conditions. In the two cases presented in this article one has been diagnosed with bipolar disorder and the other with schizophrenia.

The diagnosis of schizophrenia includes a patient exhibiting delusions, hallucinations, and/or disorganized thought/speech as well as negative symptoms [3]. Auditory and visual hallucinations (AVH) have long been a staple in the diagnosis of schizophrenia with 50 to 60 percent of individuals exhibiting AVH [2]. This makes AVH a particularly important symptom to recognize. The diagnosis is largely based on the patient's ability to verbalize these hallucinations, particularly auditory hallucinations to the clinician.

In contrast, bipolar disorder takes into account mood fluctuations over a certain period of time. These fluctuations must meet criteria for mania or hypomania depending on the type of bipolar disorder being considered. Mania is an elevation in mood with the patient displaying distractibility, indiscretions, grandiosity, flight of ideas, activity increase, decreased need for sleep, and talkativeness. It is important to obtain both collateral of the patient's history and rely on current presentation for a proper diagnosis [3].

When addressing the aforementioned diagnoses, it is imperative to think of differentials. Deaf individuals tend to be more expressive in their gestures. Again sign language is their primary form of communication. While a hearing individual may scream when angry, a Deaf person will sign aggressively. The way the Deaf culture experiences and connects with the world is foreign for those without exposure to this group [4].

## 2. Case #1

The first case concerns a 26-year-old prelingually Deaf male, with a prior history of Tourette's syndrome, bipolar disorder, and HIV, who was placed under a Baker Act at a local hospital for “acting erratic and psychotic.” A Baker Act is a 72-hour involuntary psychiatric hold within the state of Florida that can be initiated by healthcare professionals and police officers in the event of a patient being a danger to self or others. The preliminary diagnosis on the involuntary form, as per the emergency room physician, was “psychosis.” The patient was subsequently given an emergency treatment order of intramuscular lorazepam and was transferred to a psychiatric hospital where he was observed by nursing as “calm and nonthreatening.”

Prior to initial psychiatric interview, an ASL-interpreter was called to assist. The patient asked where he was at and became angry after discovering the truth of his hospitalization. He reported he initially came to the hospital as he had been having anxiety and physical pain attributed to his Tourette's Disorder. He reported his neurologist had him on carisoprodol and diazepam to help relieve these symptoms, but that they were stopped one month prior. The family was called and stated there was questionable abuse of medications but they were adamant that he was safe for himself and others.

When the patient was seen by the ED physician initially there was no interpreter present. The patient reported becoming frustrated and was trying to sign aggressively which he believes was misinterpreted. He also expressed in spoken word to the staff there that he had been “hearing voices” secondary to his pain level. He purportedly was never told what was occurring prior to seeing the interpreter at the transfer facility nearly 12 hours later. The patient adamantly denied SI, HI, AVH, or mania and maintained a linear and coherent thought process. He expressed a history of bipolar disorder which had been diagnosed after a similar incident in the past. He had been on several antipsychotics previously but had not taken any for several years without incident. He had only been taking anxiolytics and pain meds for multiple years which he felt stable on, as well as antiretrovirals for his HIV diagnosis.

The patient later admitted that he had been buying oxycodone off the street since his neurologist had stopped prescribing medications due to questionable abuse. A clinical opiate withdrawal scale was performed and was only positive for minor anxiety elevation. A full medical workup was performed and excluded any medical causes to his admission. Through further interview, OCD was excluded as a diagnosis but substance use disorder remained high on the differential for his current and past behavior. The patient was kept overnight for observation and discharged the next morning following positive report from staff. He was given extensive education on substance use as well as coping strategies to prevent readmissions. Upon discharge “unspecified psychosis” was given as his diagnosis.

## 3. Case #2

The second case involves a 30-year-old Deaf, Hispanic male who presented to the Emergency Department after his mother reported that the he was behaving oddly and not taking his risperidone. Per reports, the patient was talking to his mother about going places in a UFO and exhibiting disorganized and illogical behaviors. He was subsequently placed under a Baker Act by the emergency room physician who documented that the patient was exhibiting auditory hallucinations. Initially an interpreter was brought to the hospital prior to his admission. Per the ASL-interpreter, the patient stated that he felt “fine and not crazy” and that all of these events are happening because his mother does not “understanding Deaf culture.” He also conveyed that he did not like to take his meds because they interfered with him being able to drink alcohol and caused drowsiness.

**Figure 1:**
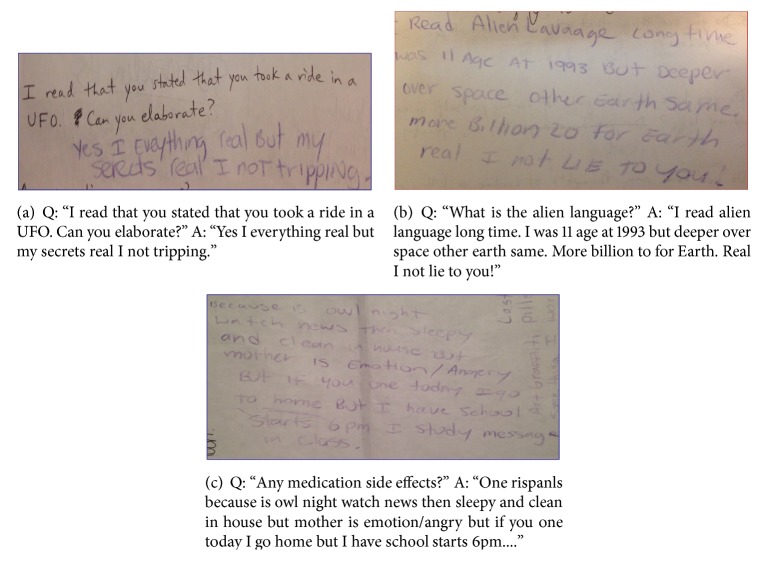


Upon initial psychiatric interview an interpreter was not present as the hospital only agreed to set periods of time for the interpreter. As an effort to communicate, questions were prepared for the patient to answer via written responses.  Figure 1 highlights a portion of the questions and answers that were constructed. From the responses he maintained bizarre delusions but denied current SI, HI, or AVH. When the ASL-interpreter arrived, the patient appeared jovial and yearned to express himself. The interpreter stated she had difficulties reading his rapid signing at first and had to have him slow down several times. However she did note that this was a common occurrence when addressing Deaf individuals.

With the interpreter's assistance, the patient was answering questions logically with a linear thought process. He reported that he had been diagnosed with schizophrenia as a teenager after having several interpersonal issues with his mother. She is Spanish speaking only and he stated that she has never fully understood how to communicate effectively with him. He had been taking risperidone for several years but was tired of continuing with the medication due to the side effects of drowsiness and weight gain, which he was never able to fully discuss with his psychiatrist. Patient reported he was in an ASL school and learning a career in massage therapy. After meeting a girlfriend there he began to develop a sense of independence that he reported his mother disapproved of. This caused an altercation that he reports his mother misinterpreted which precipitated his admission.

The patient continued to express that he was abducted by aliens as a child and could understand their language, but besides this he expressed no other psychotic processes. He was observed for two days without medications and remained calm/cooperative but was unable to participate in most activities due to limitations of the interpreter availability. After a family session was completed the patient was discharged home with plans to follow up with his community psychiatrist. The patients' diagnosis was changed to delusional disorder upon his discharge.

## 4. Discussion

The rate of psychosis between Deaf and hearing patients is thought to be approximately equal. Furthermore, the rate of psychosis diagnosed in Deaf patients by American Sign Language- (ASL-) illiterate physicians has been shown to be greater than the rate diagnosed by ASL-literate physicians. This discrepancy is thought to be due both to elements of Deaf behavior and culture (e.g., subvocal thought and language dysfluency), as well as to ASL-interpreter variables that may lead to misinterpretation of “Deaf behavior” as psychotic [2, 5]. This factor was evident in case #1 as the patient's frustrations and rapid signing was seen as manic behavior. The patient was seen for several hours without an interpreter and decisions were made prior to obtaining a full history of present illness. This not only addresses an issue with a lack of hospital policy but also one of communications. However in a published case report, rapid signing was used as a way of observing whether valproate was being efficacious for a Deaf patient's manic state [6]. This lends further need into research for this special population within psychiatry.

The phenomenon of language dysfluency makes evaluating a Deaf patient difficult even for culturally competent ASL-literate physicians and often leads to greater lengths of stay amounting to be double that of hearing patients for various reasons including deficiencies in hospital services [7]. Language dysfluency is essentially a lack of proficiency in any particular language, primarily due to early language deprivation. The risk of language deprivation remains high in Deaf patients because few hearing parents become proficient signers early on, and thus most Deaf children are not immersed in sign language until they begin their education [5]

Concerning “auditory” hallucinations in Deaf patients, it is proposed that prelingually Deaf patients perceive them as subvisual precepts (i.e., in the “mind's eye”) in the form of sign language or of lips moving and not an experience of sound [3, 8]. The existence of sound-based auditory hallucinations in Deaf people remains under debate in the psychiatric community. Regarding visual hallucinations, it is postulated that Deaf patients are more sensitive to visual processing and therefore will be less likely to experience this symptom [9]. As for case #2, based on the patient's behavior and writings, it appears as if the patient possesses a single, bizarre delusion related to alien language, and UFOs. The erratic signing and writing cannot be definitively attributed to psychosis because of the phenomenon of language dysfluency. Additionally the writings presented for case #2 are of limited value as they do not represent the patients' true language patterns and this is further complicated by the chance that the patient may have been thinking in Spanish [10].

In both cases, diagnoses were given prior to their hospital stay. Inpatient treatment tends to be short term, yet long-term continuity is required for a thorough diagnostic value. As in case #1, substance use could be the primary diagnosis given his history yet he was labeled as bipolar in the past. One must ask the question if insurance reasons dictated their current diagnosis versus communication barriers or both. Overall, special interview modifications and proper interpretation become essential with Deaf individuals. These include working with certified ASL-interpreters, avoiding use of written language, asking for summaries and making clear distinct topic changes [10]. When using an ASL-interpreter it is important to note that a certified Deaf interpreter may be used in conjunction for additional assistance given that these individuals are more specialized within Deaf culture and therefore yield fewer communication errors when it comes to emotions. However, anytime there are multiple individuals translating there can be a propulsion for misinterpretation.

Furthermore ADA provides some protection by requiring effective communication for Deaf people and some hospitals have begun using telecommunication to facilitate this deficit [11]. However these requirements are loose to interpretation and hospitals fail to provide 24-hour language assistance. The patient from case 2 was only provided with an interpreter while inpatient for a few hours per day which does not allow him to benefit from groups and coping skills training to the same extent as his peers. A case report from the American Journal of Psychiatry highlights the importance of interpreter services as positive outcomes were displayed for Deaf patients by incorporating a school for the Deaf with the hospital [12].

## 5. Conclusion

The aforementioned cases highlight the importance of understanding the Deaf culture in order to properly treat and diagnose Deaf patients. The Deaf population remains an under studied and underserved community which is often misunderstood. Holistic care within psychiatry relies on a mixture of medications, therapy, and self-care which is challenging to provide to Deaf patients. New research is needed for diagnostic screenings and delivery of therapies for this population as they require extensive modification for the Deaf community in a psychiatric setting. Also proper diagnoses are needed as misdiagnosis can lead to lifelong labeling. Most importantly all physicians and healthcare staff should be exposed to an effective training program addressing Deaf culture.

## Abbreviations

ADA: Americans with Disabilities Act
ASL: American Sign Language
AVH: Auditory and Visual Hallucinations
HI: Homicidal Ideation
HIV: Human Immunodeficiency Virus
SI: Suicidal Ideation
UFO: Unidentified Flying Object.
